# Integrating single cell transcriptomics and volume electron microscopy confirms the presence of pancreatic acinar-like cells in sea urchins

**DOI:** 10.3389/fcell.2022.991664

**Published:** 2022-08-19

**Authors:** Periklis Paganos, Paolo Ronchi, Jil Carl, Giulia Mizzon, Pedro Martinez, Giovanna Benvenuto, Maria Ina Arnone

**Affiliations:** ^1^ Stazione Zoologica Anton Dohrn (SZN), Naples, Italy; ^2^ European Molecular Biology Laboratory (EMBL), Heidelberg, Germany; ^3^ Institut Català de Recerca i Estudis Avancats (ICREA), Barcelona, Spain; ^4^ Genetics Department, University of Barcelona, Barcelona, Spain

**Keywords:** sea urchin, pancreas, acinar cells, morphology, evolution of cell types, scRNAseq, SBEM

## Abstract

The identity and function of a given cell type relies on the differential expression of gene batteries that promote diverse phenotypes and functional specificities. Therefore, the identification of the molecular and morphological fingerprints of cell types across taxa is essential for untangling their evolution. Here we use a multidisciplinary approach to identify the molecular and morphological features of an exocrine, pancreas-like cell type harbored within the sea urchin larval gut. Using single cell transcriptomics, we identify various cell populations with a pancreatic-like molecular fingerprint that are enriched within the *S. purpuratus* larva digestive tract. Among these, in the region where they reside, the midgut/stomach domain, we find that populations of exocrine pancreas-like cells have a unique regulatory wiring distinct from the rest the of the cell types of the same region. Furthermore, Serial Block-face scanning Electron Microscopy (SBEM) of the exocrine cells shows that this reported molecular diversity is associated to distinct morphological features that reflect the physiological and functional properties of this cell type. Therefore, we propose that these sea urchin exocrine cells are homologous to the well-known mammalian pancreatic acinar cells and thus we trace the origin of this particular cell type to the time of deuterostome diversification. Overall, our approach allows a thorough characterization of a complex cell type and shows how both the transcriptomic and morphological information contribute to disentangling the evolution of cell types and organs such as the pancreatic cells and pancreas.

## Introduction

Cell types consist of cells with common developmental origins and similar gene expression profiles that execute a specific gene regulatory program. The gene regulatory program associated to each cell type is tightly linked to its physiology and includes a periphery of terminal genes in charge of regulating its distinct morphological features and function. In this context, each distinct gene repertoire executing specific functions is uniquely deployed in that given cell type. One of the most intriguing scientific questions in the biology of organisms is how complex cell types arose during metazoan evolution and to how early the emergence of specific cell types can be traced.

Nowadays, technological advances in transcriptomics and imaging have increased the ease with which the combination of molecular fingerprint plus high-resolution morphology of cell types enable the tackling of such complex questions. For instance, single cell RNA sequencing (scRNA-seq) applied on organisms including both vertebrates and invertebrates, such as sponges, planarians, mollusks, cnidarians and echinoderms has led to the recognition of novel cell type families and the understanding of developmental and gene regulatory processes in these animals at an unprecedented level of detail (Fincher et al., 2018; Sebe-Pedros et al., 2018; Cao et al., 2019; Tabula Muris, 2020; Chari et al., 2021; Musser et al., 2021; Paganos et al., 2021; Salamanca-Díaz et al., 2022). Furthermore, single cell inventories have also allowed the reevaluation of developmental and evolutionary relationships of animals’ cell types. Cross-species comparison of the developmental program at a cell type level has resulted in the identification of hidden homologies between cell types and organs across taxa, sometimes separated by enormous evolutionary time spans. Examples of this are the identification of homologous cell types across flatworm species, such is the case of neoblasts, or differentiated metazoan cell types such as muscles and neurons; but also identified closely linked relationships between contractile and neural cells in the sponge, jellyfish (Hydra) and mouse atlases (Tarashansky et al., 2021).

An interesting case of an organ lacking resolved evolutionary history is the vertebrate pancreas. Pancreas is a multifunctional organ that bears specialized cell types responsible for food digestion and organismal homeostatic regulation. In brief, digestion relies on the production and secretion of digestive enzymes that catabolize large biomolecules such as proteins to amino acids, plus the hormones involved in the metabolism of glucose derivatives. The complex cell type composition, and the precise architectural organization of the pancreas, allows it to act both as endocrine and exocrine gland.

The exocrine pancreas partition consists of cells that are spatially organized into acini (Slack, 1995; Karpińska and Czauderna, 2022) and are responsible for the synthesis, storage and secretion of digestive (zymogen) enzymes such as carboxypeptidases, amylases, lipases as well as proteases and their ontogeny has been thoroughly described in the past (Husain and Thrower, 2009). Once mature, acinar cells produce and secrete the zymogen enzymes, through a mechanism involving the vesicle transport into the pancreatic lumen, thus connecting the pancreas with the digestive tract (Husain and Thrower, 2009). On the other hand, the endocrine function of pancreas, which is mainly the production of hormones with homeostatic function such as insulin, glucagon and pancreatic polypeptide (PP), is executed by diverse populations of endocrine cells that in mammals are grouped into islets (islets of Langerhans). Noteworthy, both mammalian exocrine and endocrine glands are in direct contact with a ductal epithelium, whose role is to neutralize those enzymes (Karpińska and Czauderna, 2022).

Interestingly, although the cellular composition of pancreas is widely conserved among vertebrates, the spatial distribution of the different cell types varies across taxa. For instance, a specific feature of mammalian clade is that the pancreas contains different endocrine cell types with diverse functions (α-cells, β-cells, δ-cells, ε-cells and pancreatic polypeptide producing cells), which are clustered together in the islets of Langerhans, embedded within the same tissue as acini, a specific feature of mammals (Mastracci and Sussel, 2012). In other vertebrates, such as teleost fish, the organization of the pancreatic cell types appears to be rather simple, with all cells being grouped in pancreas-like organs consisting of endocrine cells organized also in islets, but here not embedded within the exocrine tissue (Falkmer et al., 1985; Yui and Fujita, 1986; Youson et al., 2006). The formation of a distinct pancreatic organ (with different cellular architectures) seems to be a vertebrate innovation as no such structure has been so far identified in invertebrates or even non-vertebrate chordates. However, gene expression studies identified the presence of distinct cell types employing a pancreatic genetic program. However, since these animals lack pancreas as a distinct organ, they are referred to as pancreatic-like cell types. For instance previous studies have shown that while no pancreas is present in cephalochordates and tunicates, several pancreatic-like cell types are there, dispersed throughout their digestive tract (Reinecke and Collet, 1998; Olinski et al., 2006; Arda et al., 2013; Lecroisey et al., 2015). Moreover, pancreatic-like cell types have been identified even in cnidarian species suggesting that a pancreatic-like molecular machinery might have been present in the last common ancestor of cnidarians and bilaterians (the Nephrozoa). In fact, scRNA-seq of *Nematostella vectensis* revealed the presence of several genes encoding for secreted digestive enzymes that were restricted to the pharynx cnidoglandular tract (Sebe-Pedros et al., 2018). Furthermore, a similar spatial distribution of exocrine and insulin-producing cells has been reported in ectodermally-derived cell types of the sea anemone (Steinmetz et al., 2017). All the previous evidence points toward the existence of a regulatory program in the common ancestor of the Nephrozoa, which is instantiated as a fully organized pancreas only in the vertebrates.

The molecular decisions, developmental pathways, and gene regulatory networks of all stages of pancreas development have been reconstructed in great detail in various taxa, although most of the available data come from studies carried out exclusively in mammals. Such studies have highlighted the important role of several transcription factors in the specification of pancreatic progenitors, those that gives rise to both endocrine and exocrine lineages. In addition, distinct terminal differentiation gene batteries have been identified. Functional analysis has corroborated the specific role of some regulatory genes in the pancreas formation. For instance, the silencing of either the *pancreatic and duodenal homeobox 1 (Pdx1)* or the *pancreas-specific transcription factor 1a (Ptf1a)*, which are amongst the first transcription factors to be activated within the primitive murine gut tube (Burlison et al., 2008), leads to pancreatic agenesis (Ahlgren et al., 1996; Kawaguchi et al., 2002; Marty-Santos and Cleaver, 2016). Both transcription factors have been found to have a dual role, both in the initial steps of pancreatic progenitor specification and in the cell type’s differentiation. In particular, *Ptf1a* is known as essential for the differentiation of acinar cells (Zecchin et al., 2004), while *Pdx1* is re-utilized later in development to promote specifically β-cells differentiation (Macfarlane et al., 1999; Afelik et al., 2006). The tightly modulated serial activation of different transcription factor regulatory modules will give rise to distinct pancreatic types. For instance, the differentiation of β-cells depends on a gene regulatory core consisting of the transcription factors *Pdx1, NeuroD, Islet1, Nkx2.2, Pax6, Mnx, Rfx6*, and *Rfx3* with the silencing of any of those genes resulting in the impairment of β-cell maturation and function (Ait-Lounis et al., 2010; Gu et al., 2010; Gosmain et al., 2012; Ediger et al., 2014; Piccand et al., 2014; Pan et al., 2015; Gutierrez et al., 2017). In contrast, the transcription factor *Brn4* is the dominant regulator of glucagon expression in another endocrine cell population, the α-cells (Hussain et al., 2002). Here, Notch signaling has been found to promote exocrine fate through the activation of *Ptf1a, Mist1 and Rbpj* and the repression of *Ngn3* (Pin et al., 2001; Fujikura et al., 2007).

Among deuterostomes, echinoderms, in particular sea urchin larva is a unique model to address pancreas evolution and the origination of the vertebrate structure. Their phylogenetic position as non-chordate deuterostomes, the thorough characterization of their cell lineages, the availability of genomic resources and the presence of resolved at a great detail gene regulatory networks make sea urchins suitable for addressing the question of organogenesis evolution. In the larvae of the sea urchin *S. purpuratus*, cells located in the upper part of the larval stomach co-express a subset of typical pancreatic digestive enzymes. Their gene regulatory program depends on the Notch signaling pathway and the activation of the transcription factor *Sp-Ptf1a* (Perillo et al., 2016) and due to their gene expression similarities to the mammalian exocrine pancreas cells were annotated as exocrine pancreas-like cells. Furthermore, it has been demonstrated that apart from the gene expression conservation, the sea urchin homolog of the vertebrate *Ptf1a* gene can substitute for its vertebrate homolog in activating downstream gene targets (Perillo et al., 2016). In addition to the exocrine pancreas-like cells, sea urchin larva also contains specialized gut cells that produce a structurally similar protein to that of the cephalochordate amphioxus insulin-like peptide (Perillo and Arnone, 2014). Interestingly, the insulin positive cells were found in the intestinal region of the larva, which is known to be patterned and controlled by the sea urchin homolog of the vertebrate *Pdx1* gene (Cole et al., 2009; Annunziata et al., 2014). It is important to stress out that previous studies from our group demonstrated that sea urchin could potentially represent an intermediate stage in pancreas evolution, since the larva contains, apart from the endodermally-derived endocrine and exocrine-like cell types (Perillo et al., 2016). Moreover we recently provided evidence that sea urchin larvae possess an additional neuronal population with a pancreatic-like gene toolkit, whose differentiation is *Pdx1*-dependent, and was probably co-opted by modern endocrine pancreatic cells (Paganos et al., 2021). This scenario is also supported by the molecular and functional similarities of mammalian neurons and β pancreatic cells (Arntfield and van der Kooy, 2011).

Despite our previous knowledge, little is known of these pancreatic-like cell types present in the sea urchin digestive tract. There are still some open questions regarding the number of pancreatic-like cell types that need to be resolved, in particular, whether they are endocrine or exocrine-like. Additionally, it is still not clear to which extent the molecular similarity also reflects a morphological resemblance with the vertebrate pancreatic cells. In order to address these questions, we undertook a thorough molecular and morphological characterization of those cell types. Here we demonstrate that the pancreatic signature is present in distinct populations of cells within the larval digestive tract, supporting the hypothesis that the cell types constituting the pancreas are already present in a deuterostome and prior to the late coalescence in a distinctly organized multifunctional unit, the pancreas. Furthermore, we characterize molecularly and morphologically an exocrine cell type that is present in the upper part of the larval stomach, and whose specification is remarkably guided by a specific gene regulatory network used in the differentiation of mammalian pancreatic progenitors of acinar cells. Moreover, we provide evidence that the sea urchin acinar-like cells are homologous to the building blocks making the pancreatic acini in mammals. This was estimated by their molecular signature as identified using single cell transcriptomics and their distinct morphological features as shown by volume electron microscopy paired with confocal microscopy.

## Materials and methods

### Animal husbandry and larval cultures

Adult *Strongylocentrotus purpuratus* individuals were collected from the San Diego coast by Peter Halmay and shipped by Patrick Leahy (Kerckhoff Marine Biological Laboratory, California Institute of Technology, Pasadena, CA, United States). Sea urchins were housed in circulating seawater and temperature controlled aquaria at both the Stazione Zoologica Anton Dohrn, Naples, Italy and the European Molecular Biology Laboratory, Heidelberg, Germany. Gametes were collected after *in vitro* induced spawning of the adult individuals; oocytes were fertilized, and embryos/larvae were reared at 15°C in FSW diluted 9:1 with distilled H_2_O (Filtered seawater) until the developmental time-points of interest. Larval cultures were maintained by exchanging half of the FSW with fresh FSW) two times per week. After the 3 days post fertilization (dpf) pluteus stage, the larvae were fed three times per week with the unicellular micro-algae *Dunaliella* sp at an approximate concentration of 1,000 cells/mL.

### Fluorescent *in situ* hybridization and immunohistochemistry

Fluorescent *in situ* hybridization (FISH) was performed as described in Paganos et al. (2022). Embryos and larvae were fixed in 4% PFA in MOPS Buffer overnight at 4°C, washed with MOPS buffer and then stored in 70% ethanol at −20°C. Antisense mRNA probes against the genes of interest were generated as described in Perillo et al. (2021). Probes for *Pdx1, Cdx, Brn1/2/4, ManrC1a* were generated as described in Annunziata and Arnone (2014); for *Fgf9/16/20* and *SoxE* as described in Andrikou et al. (2015); for *Cpa2L* and *Ptf1a* as shown in Perillo et al., 2016; for *Islet* as described in Perillo et al., 2018 and for *Rfx6* and *Serp2/3* in Paganos et al., 2021. Primers used for the amplification of *Rfx3, Mnx, FoxA* and *Trypsin 2* can be found in Supplementary Table 1. Briefly, genes of interest were isolated from a pool of cDNAs, and the amplified products were sequenced. Probes were generated through *in vitro* transcription using Digoxigenin or Fluorescein RNA labeling mix solutions (Roche) and signal was developed through cyanine based signal amplification (Akoya Biosciences). Immunohistochemistry (IHC) was carried out as described in Perillo et al., 2021 with minor modifications. Once FISH procedure was completed specimens were placed in blocking solution containing 1 mg/ml Bovine Serum Albumin (BSA) and 4% sheep serum in MOPS buffer for 1 h at room temperature (RT). Primary antibodies were added in the appropriate dilution and incubated for 1 h and 30 min at 37°C. Endo1 (gift from Dr. David McClay) was used to mark the mid and hindgut domains (undiluted). Specimens were washed with MOPS Buffer (five times) and incubated for 1 h with the appropriate secondary antibody (AlexaFluor) diluted 1:1,000 in MOPS buffer. Samples were washed several times with MOPS buffer and imaged using a Zeiss LSM 700 confocal microscope.

### X-ray micro computed tomography

EM prepared samples (3 dpf *S. purpuratus* larvae) fixed in 2% Glutaraldehyde in FSW and embedded in resin were manually trimmed with a razor blade to reduce the size and the amount of empty resin around the larvae. When necessary, further trimming with an ultramicrotome (Leica UC7) and a diamond trimming knife (Cryotrim 90, Diatome) was done to smoothen the surfaces of the blocks and reduce potential imaging artifacts. The obtained samples were then imaged using a Bruker Skyscan 1272, using the X-ray source at a voltage of 50 kV and 200 µA current. The samples ware rotated 180° on the stage and images were collected every 0.2° with an exposure time of 519 ms at 1 µm voxel size. The 3D volume was finally reconstructed using the software NRecon (Bruker).

### Volume electron microscopy

*S. purpuratus* 3 dpf larvae were frozen at a high pressure with an HPM010 (Abra Fluid) high pressure freezer using 20% Ficoll 70 (Sigma) in FSW as a cryoprotectant. The samples were then freeze substituted with a solution of 1% OsO_4_, 0.5% Uranyl Acetate and 5% H_2_O in acetone, using a Leica AFS2. They were left afterwards for 64 h at −90°C before increasing the temperature to −30°C using a speed of 5°C/h. After 4 h incubation at −30°C the temperature was gradually increased to +20°C (5°C/h), and the samples were further incubated for 5 h. After this, samples were manually rinsed with dry acetone before putting them in a solution of 0.1% Thiocarbohydrazide and 10% H_2_O in acetone for 20 min at room temperature. The contrast was then further increased by a second OsO_4_ incubation (1% in acetone). Infiltration in Durcupan (Sigma) was performed with a Pelco Biowave (Ted Pella). The samples were flat-embedded, and the resulting blocks were scanned with an X-ray micro computed tomography apparatus [Skyscan 1272, Bruker, referred to as micro computed tomography (microCT)] in order to identify and target the best preserved larvae. The sample was then trimmed (UC7, Leica) using a 90° cryo-trimmer (Diatome) to generate a small block face (∼400 μm × 400 µm) and to approach in depth the larva of interest. The resulting resin block was mounted on a pin stub using silver conductive epoxy resin (Ted Pella). The serial block face acquisition was performed with a Zeiss Gemini2 equipped with a Gatan 3view microtome and a focal charge compensation device (Zeiss). The SEM was operated at 1.5 kV 300 pA, using a pixel size of 15 nm, a dwell time of 0.8 µs and a slice thickness of 50 nm. The acquisition of the full larva images was performed using the software SBEMimage (Titze et al., 2018). After acquisition, alignment of the sections and the tiles containing the volume of interest (covering the larva stomach) were aligned and blended using the FiJi plugin TrackEM2 (Cardona et al., 2012). Segmentation and analysis were performed using the software package Amira (Thermo Fischer Scientific). The aligned SBEM dataset was imported in a x,y binned form to identify regions of interest, which were then imported at full resolution for further study. Nuclei, vesicles and plasma membranes were segmented in a semi manual fashion using the thresholding and Magic Wand tools in Amira. The resulting segmented objects were further rendered for three-dimensional visualization using the surface rendering module and analyzed using the label analysis module to quantify organelle volumes and positions. Vesicles were classified as apical based on their position below the base of the nucleus in direction of the apical plasma membrane.

### Single cell RNA sequencing analysis

The Single cell RNA data analyzed in this study originate from the study by Paganos et al. (2021) as they have been deposited in Dryad under the unique identifier https://doi.org/10.5061/dryad.n5tb2rbvz. Subclustering analysis has been performed as described in Paganos et al. (2021) using the Seurat scRNA-seq R package (Butler et al., 2018; Stuart et al., 2019). Subclustering analysis was performed by selecting and subsetting the cell type families of endodermal origin (esophagus, cardiac sphincter, exocrine pancreas-like, stomach 1, 2, and 3, pyloric sphincter, intestine and anus). Datasets were normalized and variable genes were found using the vst method with a maximum of 2,000 variable features. These datasets were scaled, and principal component (PCA) analysis was performed. Nearest Neighbor (SNN) graph was computed with 20 dimensions (resolution 1.0) and Uniform Manifold Approximate and Projection (UMAP) was used to perform clustering dimensionality reduction. Cluster markers were found using the genes that are detected in at least 0.01 fraction of min.pct cells. The average score of pancreatic gene markers was estimated using the AddModuleScore function incorporated in Seurat R package. The presence of transcripts per cluster was visualized using the DotPlot or DoHeatmap functions incorporated in the Seurat package.

## Results

### Pancreatic-like signature across larval cell type families

**FIGURE 1:**
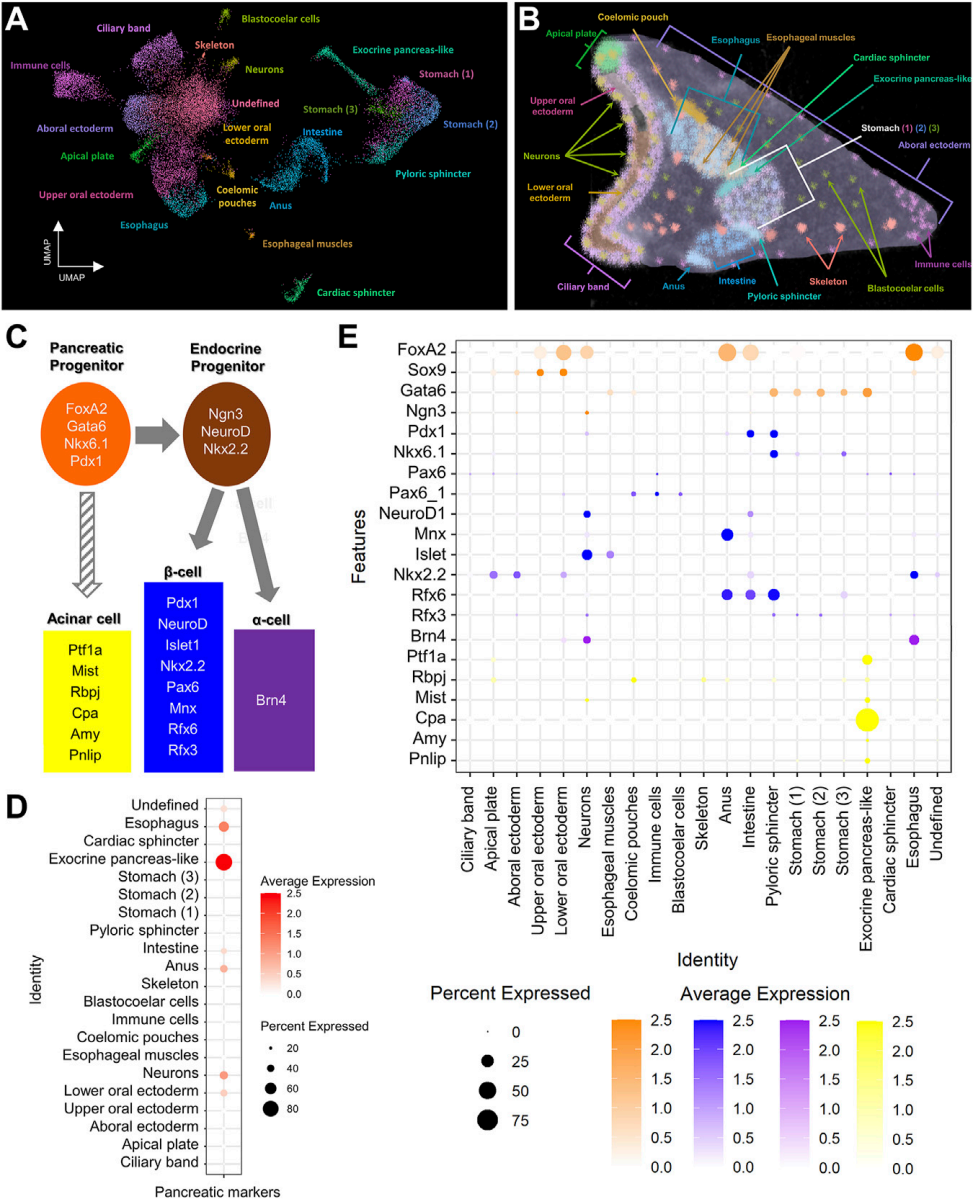
Pancreatic-like molecular signature of the *S. purpuratus* larva cell type families. **(A)** Uniform Manifold Approximation and Projection (UMAP) showing the 3 dpf *S. purpuratus* cell type families (modified from Paganos et al., 2021). **(B)** X-ray Microtomography (MicroCT) of the 3 dpf *S. purpuratus* pluteus larva, placed in lateral view, in which the different cell type families are labelled with pseudo-coloring. Color code is the same as the one used in **(A)**. **(C)** Simplified schematic representation of pancreatic gene markers known to be present in specific stages of pancreatic development and distinct pancreatic lineages. **(D)** Dotplot showing the average score of the sea urchin pancreatic marker orthologs shown in **(C)** across the larval cell type families. **(E)** Dotplot showing the average expression of the pancreatic markers across the larval cell type families. Color code in **(E)** is the same as the one used in **(C)**.

Single cell RNA sequencing has been shown to be a great resource in analyzing fine developmental events as well as identifying complex molecular signatures during sea urchin embryogenesis (Massri et al., 2021; Paganos et al., 2021). Previously, we have used single cell RNA sequencing originating from 3 dpf *S. purpuratus* larvae to reconstruct the cell type family composition of the *S. purpuratus* pluteus larva (Paganos et al., 2021). The results of that analysis resulted in the identification of 21 cell type families with diverse regulatory signatures (Figures 1A,B) as well as 12 distinct neuronal types, one of which was found to utilize, surprisingly, a gene regulatory toolkit that is present in mammalian endocrine cells. Based on those findings, confirming the presence of neuroendocrine sea urchin cells that employ an evolutionary conserved pancreatic gene regulatory program led by *Pdx1*, we decided to investigate whether a pancreatic-like molecular signature is deployed in other sea urchin larval cell type families. To do so, we selected, as guides, a core (module) of typical mammalian transcription factors that are known to be essential for pancreatic cells specification and/or differentiation (Figure 1C). Plotting for the average score of the sea urchin orthologs of these markers reveals an enrichment of this module in ectodermally-derived upper oral ectodermal and neuronal cell type families as well as in endodermally-derived cells corresponding to anal, intestinal, exocrine pancreas-like and esophageal clusters (Figure 1D), all suggesting a broad use of this module in a variety of larval cell types. Similar data were obtained by plotting for the average expression of each member of this module highlighting the expression of endocrine pancreas gene markers in both neurons (*FoxA2, Ngn3, Pdx1, NeuroD1, Mnx, Islet, Rfx3, Brn*4 and *Mist*) and intestinal cells (*FoxA2, Pdx1, NeuroD1, Mnx, Nkx2.2* and *Rfx6*), thus suggesting a shared pancreatic regulatory wiring in these two cell type groups (Figure 1E). Interestingly, the same plot reveals the presence of an endocrine pancreas regulatory wiring also in the pyloric sphincter cell types expressing *Gata6, Pdx1, Nkx6.1, Rfx3* and *Rfx6* genes. Moreover, these data, taken together, suggest the presence of at least two distinct endocrine and exocrine cell fates, with the endocrine deployed in both ectodermally- and endodermally-derived cell type families, and the exocrine limited to a well-defined cluster (Figures 1D,E).

### Assessing the pancreatic signature of the digestive tract at a single cell resolution

**FIGURE 2:**
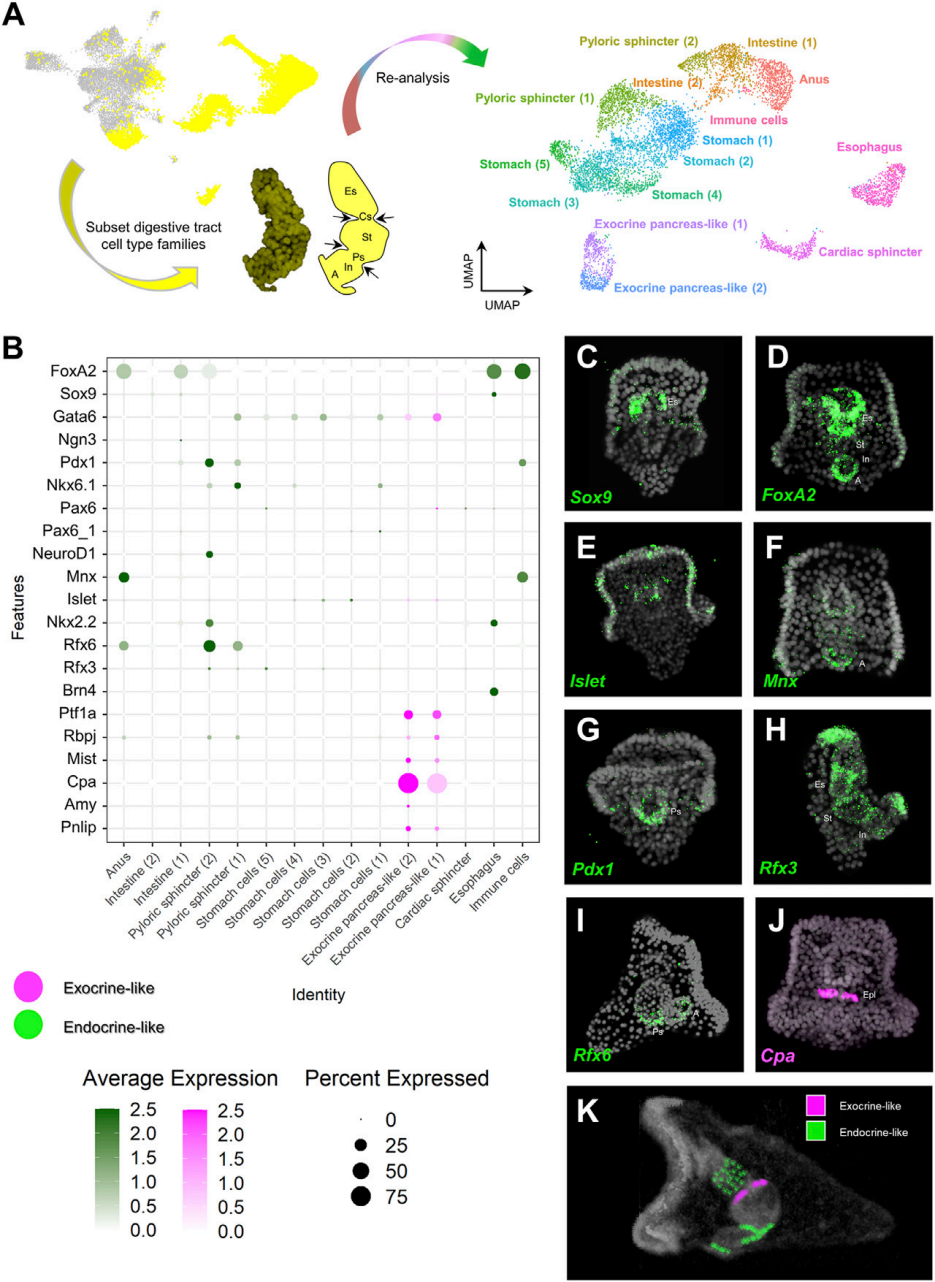
Distribution of endocrine and exocrine pancreas-like markers across the digestive tract. **(A)** Summary of the pipeline used to increase the resolution of the digestive tract cell type composition. Digestive tract related cell type families are highlighted in yellow (left), subsetted and subclustered resulting in the generation of 15 clusters (right). **(B)** Dotplot showing the average expression of pancreatic gene markers across the digestive tract subclustered dataset. Colored boxes are used to highlight endocrine-like (green) and exocrine-like (magenta) molecular signatures. **(C–J)** FISH using antisense probes designed against several of the pancreatic markers used in **(B)**. Color code of the signal is the same as in **(B)**. DAPI was used to label the nuclei (gray). **(K)** MicroCT of the 3 dpf *S. purpuratus* pluteus larva, placed in lateral view, in which the different pancreatic-like molecular fingerprints found in the digestive tract are labelled with pseudo-coloring. Color code is the same as the one using in **(B)**. A, Anus; Es, Esophagus; Cs, Cardiac sphincter; Epl: Exocrine pancreas-like; In, Intestine; Ps, Pyloric sphincter; St, stomach.

In order to further investigate the pancreatic-like signature of the digestive tract at a higher resolution, we performed subclustering analysis solely of clusters representing endodermally-derived cell type families. This analysis, originating from nine initial clusters resulted in the generation of 15 distinct subclusters (Figure 2A) for which the marker genes were extracted (Supplementary File 3). Next, we used the marker genes in combination with already known lineage and cell type specific gene markers (Supplementary Figures S1A,B to annotate them. For instance, such markers included the transcription factors *Sp-Bra* and *Sp-Hox11/13b* known to be expressed in cells located in the anal region of the digestive tract (Annunziata et al., 2014; Paganos et al., 2021), *Sp-Ffg9/16/20* expressed in the anal, pyloric and cardiac sphincters domains (Paganos et al., 2021), *Sp-ManrC1A* which is a midgut/stomach molecular marker (Annunziata et al., 2014; Paganos et al., 2021), and *Sp-Brn1/2/4* expressed specifically in the esophageal domain (Cole et al., 2009; Perillo et al., 2018; Paganos et al., 2021). Interestingly, we detected the presence of an unexpected cluster composed of cells that, based on their molecular signature, correspond to an anal subtype. These cells appear to also co-express genes related to the mesodermally-derived lineage of pigment cells and were therefore annotated as immune cells. Sea urchin pigment cells are immune cells known to migrate from the tip of the archenteron to the ectoderm during gastrulation and from the ectoderm to the digestive tract later on in development and the presence of pigment cells in the anal domain have been validated by previous studies (Buckley and Rast, 2017; Perillo et al., 2020). Whether the cells we find in our digestive tract subclustering correspond to transfated pigment cells, that employ an endodermal genetic program after migration to the anal region or endodermally derived pigment cells needs further investigation.

Once the identity of the subclusters was validated, we set out to explore the expression of specific pancreatic markers in the different digestive tract-associated cell types. Plotting for the average expression of these genes validated our initial assessment of a broad endocrine-like signature in a plethora of digestive tract clusters and the confined expression of exocrine pancreas genes in only two very similar subclusters (Figure 2B). Specifically,

The validation of the single cell predictions was done through the use of FISH and using specific antisense mRNA probes against the sea urchin orthologs of several key pancreatic markers. The FISH data confirmed the shared expression of the sea urchin orthologs of *Sox9* and *FoxA2* (Figures 2C,D) in the esophageal region, the neuronal expression of *Islet* (Figure 2E), the cell type specific expression of *Cpa* (*Sp-Cpa2L*) in the upper part of the midgut region (Figure 2J), the broad expression of *Rfx3* across the digestive tract cell types (Figure 2H) and the enrichment of *Mnx*, *Pdx1* and *Rfx6* in cell populations of the posterior gut (Figures 2F,G,I, respectively). The results of this analysis are summarized in Figure 2K.

### Molecular characterization of the sea urchin exocrine pancreas-like cells

**FIGURE 3:**
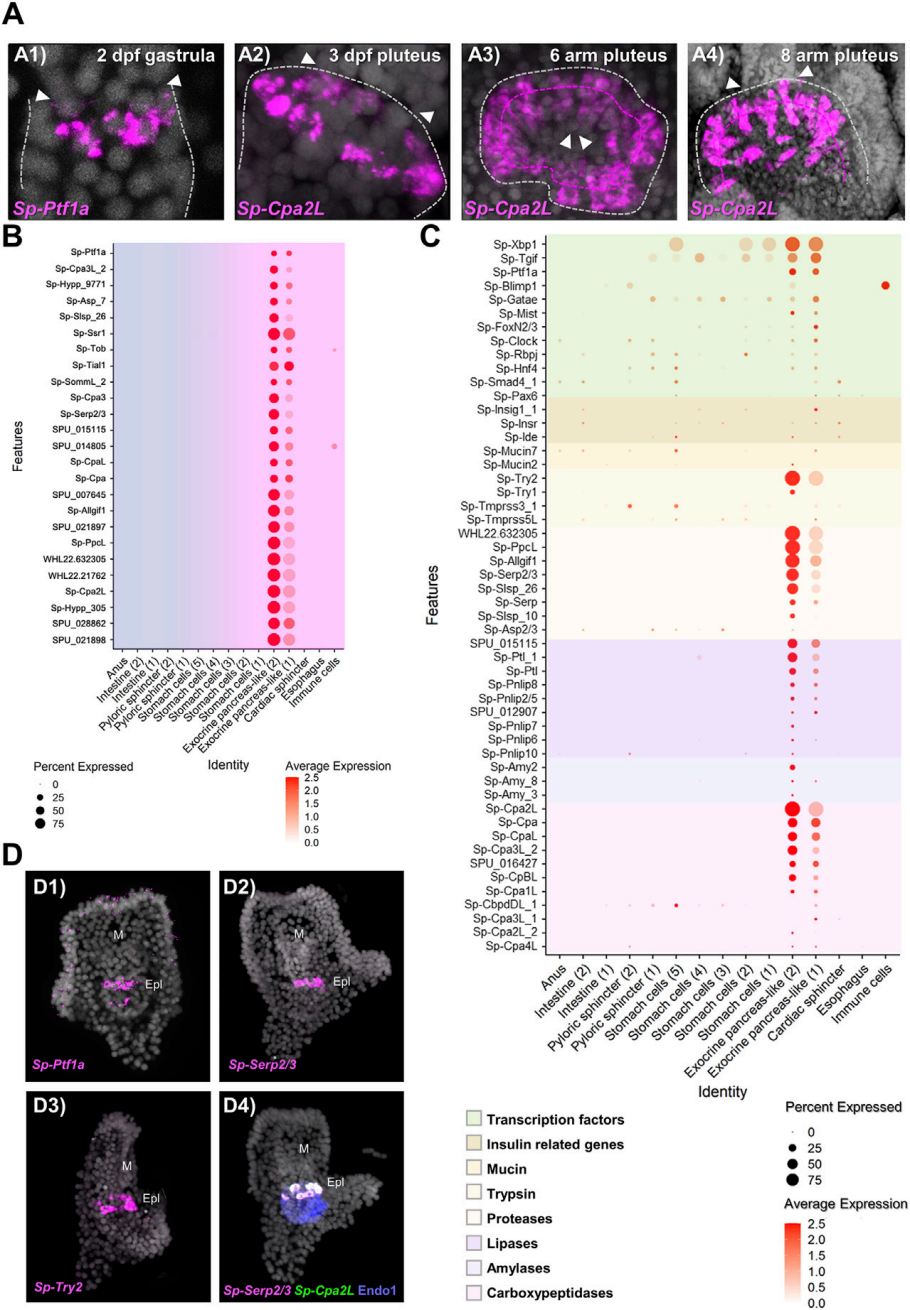
Molecular characterization of the *S. purpuratus* larva exocrine pancreas-like cells. **(A)** FISH using antisense probes against *Sp-Ptf1a* (A1) and *Sp-Cpa2L* (A2–4) at gastrula and various larval stages, respectively. Contour of the digestive tract compartment (gray) and the double belt row structure (magenta) are shown in dotted lines. Arrowheads indicate the position of the presumptive (A1) and the actual cardiac sphincter (A2–4). Specimens in A1, A2, and A4 are oriented in lateral view and the larva in A3 is viewed from the cardiac sphincter. **(B)** Dotplot showing the average expression of the top 25 exocrine pancreas-like marker genes. **(C)** Dotplot showing the average expression of sea urchin orthologous genes encoding for proteins found in mammalian acinar cells. **(D)** FISH validations of gene predictions shown in **(C)** using antisense mRNA probes for *Sp-Ptf1a* (D1), *Sp-Serp2/3* (D2, D4), *Sp-Trypsin2* (D3) and *Cpa2L* (D4). FISH shown in D4 was paired with IHC for the midgut and posterior gut marker Endo1. DAPI was used to label the nuclei (gray). Epl, Exocrine pancreas-like; M, Mouth.

Once the mapping of the pancreatic-like cell types in the different parts of the digestive tract was established, we set out to characterize in detail the exocrine pancreas-like cells, which was the primary goal of our study. Previous studies from our group demonstrated that a unique population of cells producing digestive enzymes and located in the upper part of the larval midgut. These cells use a pancreatic-like regulatory circuit that is activated by the Notch signaling and includes the sea urchin homologs of the transcription factors *Hnf1a* and *Ptf1a*, that in mammals is controlling the specification and differentiation of pancreatic acinar cells (Perillo et al., 2016). The subclustering analysis reported in this study revealed the presence of two extremely diversified clusters within the digestive tract that correspond to exocrine pancreas-like cells, as suggested by the expression of the transcription factors *Sp-Ptf1a*, *Sp-Rbpj*, plus the presence of the enzymes carboxypeptidase (*Cpa*), amylase (*Amy*) and pancreatic lipase coding genes (*Pnlip*). *In situ* hybridization using antisense probes for *Sp-Ptf1a* and *Sp-Cpa2L* corroborated our previous observations that there is a continuous increase of the number of cells constituting the exocrine cell type over time (Figure 3A). These exocrine cells originate at late gastrula stage (2 dpf) as two *Sp-Ptf1a* positive cells in the midgut domain (Figure 3, A1); at 3 dpf pluteus larva, these adopt the shape of a belt-like structure consisting of five to seven cells (Figure 3, A2) located in the upper part of the stomach. As larval development continues, a second tier of exocrine-like cells arises right next to the first one (Figure 3, A3) and the double rowed belt structure is retained up to the end of the larval development, at the competent 8 arm pluteus stage (Figure 3, A4).

Based on the above results, we explored the system to understand to what extent the molecular signature of the sea urchin exocrine pancreas-like cells is similar to the one of the mammalian acinar cells. For this reason, we next investigated whether the genes encoding for proteins involved in the exocrine function in vertebrates are also expressed in the exocrine pancreas-like cells of the sea urchin. It is well known that the exocrine process depends on the coordinated function of trafficking proteins including members of the syntaxin and synaptotagmin families, proteins which promote exocytosis of cargo-loaded vesicles (Messenger et al., 2014). Here we found that the exocrine pancreas-like cell type family expressed mRNAs related to vesicle trafficking such as *Sp-Nsf, Sp-Trappc2, Sp-Sec22*, various syntaxins (*Sp-Stx5, Sp-Stx6, Sp-Stx7, Sp-Stx12, Sp-Stx18*) and synaptotagmins (*Sp-Syt6, Sp-Syt7, Sp-Syt15*), coating such as mRNAs encoding for clathrin heavy and light chains and exocytosis mRNAs encoding for different exocyst complex component proteins (Supplementary Figure S2; Supplementary File 1). The expression pattern of all of them appears to be highly restricted to this cell type family since no co-localization of these mRNAs could be observed in any other cell type family of the larva (Supplementary Figure S2; Supplementary File 1). However, traces of this molecular signature were detected in neighboring cell types such as stomach cells and the cardiac sphincter as well as the neuronal cell type family suggesting the use of a similar but not identical vesicle trafficking toolkit between neuroendocrine and exocrine cells.

To better understand how similar the exocrine cells are to the mammalian acinar cells we looked for the presence of diverse gene families including transcription factors and terminal differentiation genes that are typically expressed in pancreatic acinar cells. Regarding transcription factors, we found a cell type specific expression of a module that includes the transcription factors *Sp-Xbp1, Sp-Tgif. Sp-Ptf1a, Sp-Blimp1, Sp-Gatae, Sp-Mist, Sp-Fox2/3, Sp-Clock, Sp-Rbpj, Sp-Hnf4, Sp-Smad4* and *Sp-Pax6* (Figure 3C, D1) typical of pancreatic cells. Pancreatic acinar cells have a complex physiology and their proper function to produce and secrete zymogens is controlled by signals originating from neighboring pancreatic cell types. Plotting for genes related to one of the most important pancreatic hormones, the insulin, we found the presence of insulin related genes such as insulin receptor (*Sp-Insr*) and insulin degrading enzyme (*Sp-Ide*) in the exocrine clusters as well as two mucin genes (*Sp-Mucin2* and *Sp-Mucin7*) known to encode for mucin proteins used for protective and lubricative role in acinar cells (Figure 3C). The presence of transcriptional regulators and effector proteins supports, with confidence, the presence of acinar-type cells in the sea urchin. However, what really differentiates pancreatic acinar cells from the rest of the pancreatic lineages is their ability to produce zymogens involved in food digestion. Typical pancreatic zymogens include proteases, lipases, amylases and carboxypeptidases. Previous studies in our laboratory demonstrated the presence of at least one carboxypeptidase (*Sp-Cpa2L*), one amylase (*Amy3*) and one pancreatic lipase (*Pnlip2/5*) gene expressed in the exocrine domain of the sea urchin larva (Perillo et al., 2016). A further characterization of these genes’ domains of expression would provide evidence for the co-expression of those and additional members of the exocrine pancreatic gene families (Figure 3C,D). Based on our analysis presented in this study, *Sp-Trypsin1 (Try1)* and *Sp-Trypsin2 (Try2)* were predicted to be specifically expressed in the two exocrine populations, and here the expression of the latter was also confirmed by FISH (Figure 3, D3). Similarly, we extended our analysis to least eight additional members of the protease family and those were found to be marking specifically the exocrine cells as is the case of the protease *Sp-Serp2/3*, which was found to be expressed within those cells and co-localized with *Sp-Cpa2L* transcripts (Figure 3; D2, D4). Moreover, members of the ribonuclease and deoxyribonucleases protein families were also found to be differentially expressed in the exocrine cells (Supplementary Files 2 and 3). Overall, we were able to expand our knowledge regarding the exocrine cells specific expression of carboxypeptidases, amylases and lipases by adding the expression of 10, 2 and 8 new members, respectively (Figure 3C), while also discovering the presence of proteases and (deoxy)ribonucleases in sea urchin exocrine cells.

Next, we set out to explore how unique is the molecular signature of the two exocrine putative cell types in respect to the rest of the digestive tract cell types. Plotting of the top 25 exocrine marker genes (Figure 3B) showed a well-confined expression of these genes only in the exocrine pancreas-like clusters suggesting that: 1) a highly diversified genetic program operates in these cells; 2) cells in these two clusters express similar genes but at different expression levels, which is indicative of the presence of diverse developmental exocrine states. To further understand the nature of the two exocrine clusters we compared their transcriptomic profile. This analysis showed that the two exocrine clusters are very similar to each other and distinct from the rest of the digestive tract cell types. However, their mutual comparison revealed fine transcriptomic differences (Supplementary Figure S3A). Looking carefully into the genes that were found differentially expressed in the two clusters we found that genes that are related to a midgut cell fate such as *Sp-ManrC1A* were enriched in the exocrine pancreas-like cluster-1 (Supplementary Figure S3B). On the other hand, exocrine cells markers expressed in fully differentiated exocrine pancreas-like cells such as *Sp-Cpa2L*, *Sp-Cpa3* and *Sp-Serp2/3* were detected among the top 25 marker genes of the exocrine pancreas-like cluster-2 (Supplementary Figure S3C). Taken together, our data suggest the existence of distinct developmental states of the exocrine cells that could potentially give rise to a unique fully differentiated exocrine cell type.

### Morphological characterization of the sea urchin exocrine pancreas-like cells

**FIGURE 4:**
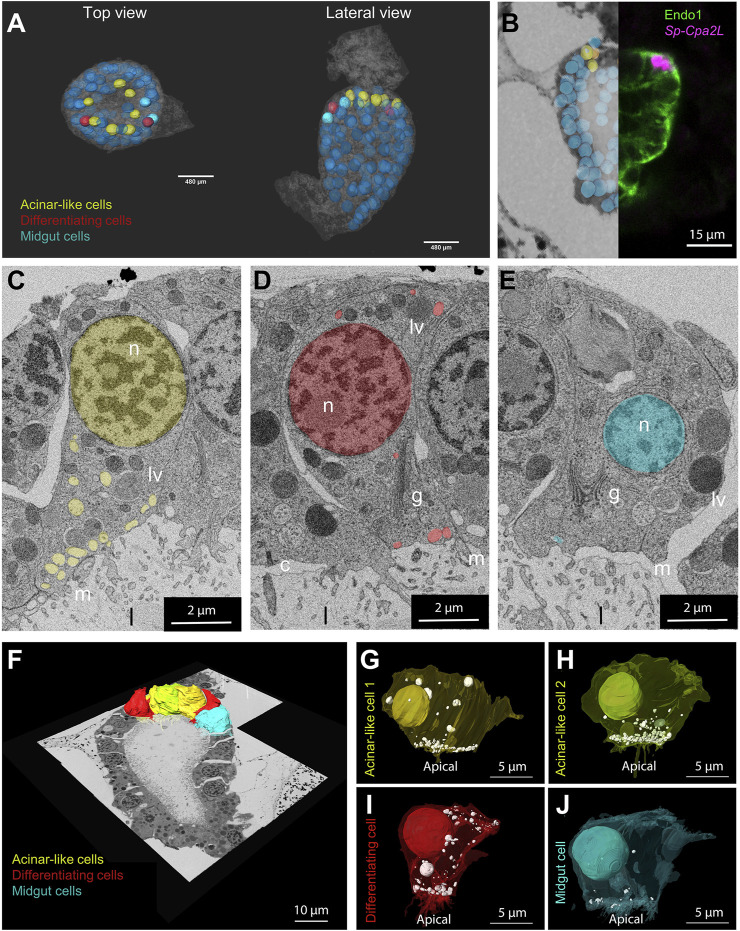
Morphological characterization of the *S. purpuratus* larva exocrine pancreas-like cells. **(A)** Nuclei segmentation of the entire midgut region in which cell membranes are visible (semitransparent). Midgut (blue), differentiating (red) and exocrine pancreas-like nuclei (yellow) are shown. The nuclei shown with lower transparency are the ones segmented and used for the analysis. Cell membranes are semi-transparent. **(B)** Overlay and stitching of the FISH for Sp-Cpa2L (magenta), IMH for endo1 and the vEM dataset. **(C–E)** Isolated slices showing an example of the vesicle relative number and polarization per cell type identified. 3D reconstruction of the cells located in the upper part of the midgut region. Plasma membranes of cell types analyzed were segmented and shown projected on the stitched EM dataset. **(F–J)** Individual 3D reconstructions of the segmented cell types of interest. The plasma membranes visible in a semi-transparent manner, revealing the nuclei and vesicle number and position. Yellow and lime, exocrine cell; Red, differentiating cell; cyan; main stomach cell. c, cilium; g, golgi; n, nucleus; l, lumen; lv, larger vesicles; m, microvilli.

The assessment of cell type evolution relies on the identification of evolutionary conserved regulatory programs that in case of homologous cell types give rise to distinct cell morphologies. Our scRNA-seq analysis applied on the 3 dpf *S. purpuratus* pluteus larva has revealed a high degree of molecular conservation between the sea urchin exocrine pancreas-like cells and the mammalian pancreatic acinar cells. Next, we pondered on the question whether their gene expression similarities are also reflected at a cellular phenotypic level. To address this question, we performed a 3D ultrastructural analysis of a 3 dpf larva using volume electron microscopy (vEM). To this aim, larvae were high pressure frozen, freeze substituted and embedded in an epoxy resin. The full volume of a characteristic larva (as judged by X-ray microCT) was finally imaged by serial block face electron microscopy (SBEM) at a resolution of 15 × 15 × 50 nm^3^ and the resulting images were computationally aligned in order to reconstruct the 3D organization of the midgut region (Figure 4A).

Manual image alignment with the FISH analysis allowed us to predict the location of the exocrine pancreatic cells in the first layer of midgut, immediately below the cardiac sphincter domain (Figure 4B). Examining the vEM dataset, we checked whether we could identify in this region cells that showed a distinct morphological signature. Interestingly, the ultrastructural analysis revealed the presence of a cluster of seven cells with specific characteristics. Because of their location and of their distinct morphological signature, we could identify them as exocrine pancreatic-like cells. Comparing them with other cells located in the midgut region, we could identify common features, as well as specific traits (Figures 4C–E). All midgut cells are polarized, with the Golgi apparatus present in the apical side, where they present a cilium and several villi protruding in the gut lumen. Moreover, they all contain a few large (∼1 µm) vesicles filled with electrondense content, which we speculate could be digested endocytosed material. However, the cluster of cells adjacent to the cardiac sphincter, are characterized by a large number of vesicles (∼300 nm in diameter) with non-electron-scattering content accumulated near the apical plasma membrane (Figure 4C). Interestingly, we also found two cells, next to the exocrine cells, in which more vesicles are present with respect to the other midgut cell, but these are less apically polarized (Figure 4D). Due to the intermediate phenotype of these cells, we believe they may be potentially differentiating cell. To confirm the characteristics of the different cell types as seen in the 2D images, we selected examples of different individuals for each class, performed a 3D segmentation and quantified the number and polarization of the light vesicles (Figures 4F–J). This analysis confirmed that the exocrine pancreas-like cells, as well as the putative differentiating cells, contain at least three times more vesicles than a typical main stomach cell (Supplementary Figure S4). Moreover, the localization of these vesicles is highly polarized towards the apical plasma membrane in exocrine cells (>75% of vesicles present between the basis of the nucleus and the apical membrane), but it is not for the putative differentiating cells (<50%; Supplementary Figure S4).

This analysis confirmed that all the exocrine pancreas-like cells contain vesicles localized in the apical part of the plasma membrane towards the lumen and that they contain at least three times more vesicles than a main stomach cell (Supplementary Figure S4). Regarding the putative differentiating cell type, we confirmed that they contain similar number of vesicles when compared to the exocrine pancreas-like cells, while they lack specific localization (Supplementary Figure S4).

## Discussion

### Pancreatic-like cells in sea urchin revealed a fundamental step in pancreas evolution

Exploring the scRNA-seq dataset of the 3 dpf *S. purpuratus* pluteus larva (Paganos et al., 2021) for a pancreatic-like molecular fingerprint resulted in the identification, and validation, of the presence of previously proposed pancreatic-like cell types in echinoderms. As previously suggested (Perillo et al., 2018; Paganos et al., 2021), the sea urchin larva nervous system utilizes a large amount of pancreatic transcription factors, which is also supported by the data of the present study. According to our analysis, the sea urchin neurons score the highest outside the endodermally-derived cell type families and utilize transcription factor orthologous to those used in most of the mammalian pancreas cell lineages, with an enrichment of the β endocrine transcription factors *Pdx1*, *NeuroD1*, *Mnx*, *Islet* and *Rfx3*, suggesting once more the tight evolutionary (and/or phenotypic) link between neural and pancreatic cells. In addition to the evolutionary conserved program between these two distal cell type families, the data suggest that sea urchin larva could potentially be an ideal candidate to address the evolution of endocrine systems.

According to our single cell data, the digestive tract of the sea urchin larva contains two major categories of pancreatic like cells: endocrine and exocrine-like. Endocrine cell markers are found in cell clusters scattered across the digestive tract, the exocrine ones, are confined in two distinct clusters of the digestive tract dataset and thus suggesting a highly diversified signature and role of the two systems. Cells of the anal, intestinal, pyloric sphincter and esophageal domains appear to have a strong endocrine signature, which is in agreement with previous data showing the expression of an insulin-like peptide that structurally is related to the cephalochordate insulin in the posterior gut of the late pluteus larva (Perillo and Arnone 2014). Exploring our data of the 3 dpf larva for the expression of the insulin gene did not produce any obvious results, in line with its known expression restricted to late stages and suggesting that the endocrine-like cells found in the posterior gut of the 3 dpf larva are probably immature. Nonetheless future studies are needed to distinguish whether these intestinal and pyloric sphincter cells reflect a distinct developmental state of the insulin producing cells or mature cells arise from cell progenitors altogether specified later during development. Overall, our analysis revealed the presence, at a single cell resolution, of endocrine and exocrine-like molecular signatures scattered in distinct cell types of the *S. purpuratus* larva. These findings are in line with previously proposed hypothesis that the emergence of pancreatic cell types happened in distinct steps during evolution and that such cell types are all present in the sea urchin.

### Homology between sea urchin and vertebrate pancreatic acinar cells

Our analysis confirms that the sea urchin exocrine cells utilize a pancreatic gene regulatory program including orthologs of the transcription factors *Ptf1a, Mist, Xbp1, Tgif, Blimp1, Gata6. Pax6, Rbpj* and *Hnf4*, all of which are well known pancreatic gene markers and most of them regulators of pancreas development (St-Onge et al., 1997; Huang et al., 2008; Husain and Thrower, 2009; Hess et al., 2011; Martinelli et al., 2013; Chiou et al., 2017). For instance, *Rbpj* is a transcription factor known to partner with *Ptf1a* to execute its regulatory function (Masui et al., 2007), while the transcription factor *Xbp1* is shown to be required for the homeostasis of acinar cells since its depletion in mice results in extensive apoptosis of those cells (Hess et al., 2011). Furthermore, we report here that sea urchin exocrine pancreas-like cells are able to synthetize proteins of the protease and trypsin families and also use an expanded set of carboxypeptidases, amylases and pancreatic lipases.

One unique characteristic of pancreatic acinar cells from different species is that they present a morphology that is highly polarized, with the secretory vesicles concentrated at the apical side of their plasma membrane, facing the lumen (Husain and Thrower, 2009). Here, using volume EM, we showed that sea urchin exocrine, pancreas-like cells contain a larger number of vesicles when compared to the rest of the midgut cells, with a similar accumulation of vesicles at the apical side of the plasma membrane, reminiscent of what is found in mammalian acinar cells. Interestingly, our integrated approach allowed us to identify, with a high degree of confidence, a population of not fully differentiated exocrine pancreas-like cells located next to the exocrine cells. The potentially undifferentiated state of these cells is suggested by: 1) the expression of more transcription factors in one [exocrine pancreas-like (1)] of the two recognized exocrine pancreas-like cell subclusters, in combination with the reduced expression of terminal differentiation genes, 2) the greater transcriptional similarity of this subcluster to the midgut domain, 3) the comparable number of vesicles to the exocrine-like cells, which are lacking subcellular polarization and 4) the demonstrated increase in sea urchin exocrine pancreas-like cell numbers and complexity during development, compatible with a continuous cellular differentiation of newly specified exocrine cells along development.

**FIGURE 5:**
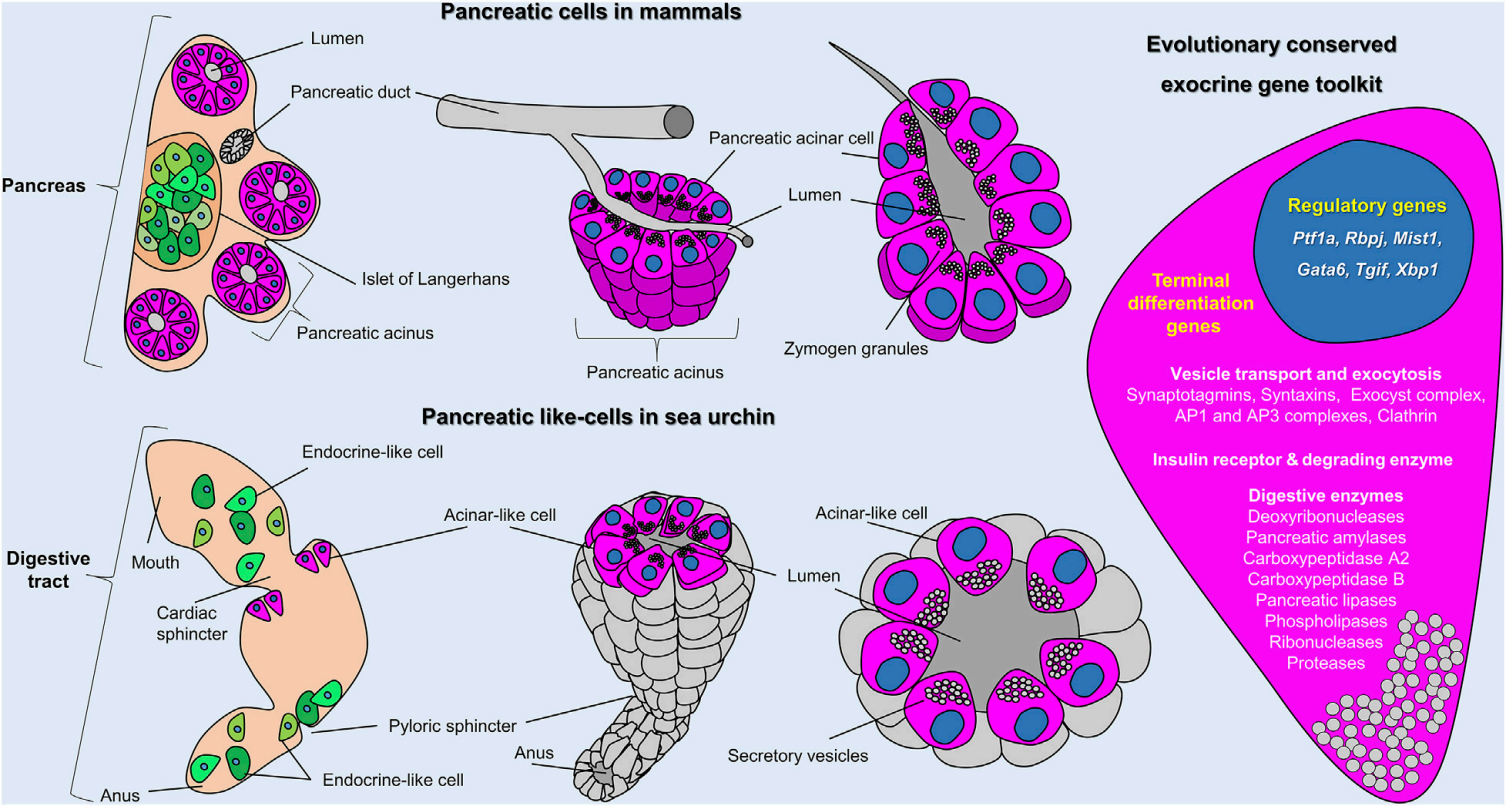
Sea urchin acinar-like cells. Schematic representation summarizing the molecular and morphological conserved features of vertebrate acinar and invertebrate acinar-like cells as shown in this study. Cartoons show the distribution and organization of pancreatic cells in mammals (top row) and sea urchin (bottom row) and (on the right) an overview of the evolutionary conserved gene toolkit shared between sea urchin and mammalian exocrine pancreatic cells. In mammals, exocrine (magenta) and endocrine (green) cells coalesce into a single organ; the pancreas. In sea urchins, acinar-like (magenta) and endocrine pancreas–like (green) cells are scattered within the digestive tract. The cartoon on the right represents an overview of the evolutionary conserved regulatory and terminal differentiation genes found both in mammalian and sea urchin exocrine cells. A full gene list of the sea urchin transcripts found in the acinar-like cell type family can be found in Supplementary Files 2 and 3.

Taken together, our results demonstrate that the sea urchin larva possesses exocrine pancreatic-like cells that we interpret as homologous to the mammalian acinar cells (Figure 5). Their homology is supported by both molecular and morphological characters, including the evolutionary conservation of the: 1) ontogenesis program, both deriving from the endoderm, 2) molecular specification pathways, 3) molecular fingerprint, 4) spatial organization, with sea urchin acinar-like cells being grouped together forming belt-like structures 5) and cells’ morphology. These lines of evidence suggest that those cell types in sea urchins and vertebrates are, thus, homologous, sharing a common ancestry. Whether this homology can be extended outside the deuterostome clade remains an open question that needs further analysis. However, we envision such a possibility since digestive cells with similar gene expression profiles have been reported in sponges and cnidarians (Steinmetz et al., 2017; Sebe-Pedros et al., 2018; Musser et al., 2021). In the latter cases we are still lacking the precise molecular and morphological data to support such an evolutionary link.

### Coalescence of cell types in a single organ (pancreas) is necessary for the efficient crosstalk between the exocrine and endocrine fractions

In conclusion, we show that the integration of different approaches is sufficient and essential for the thorough characterization of a give cell type and the assessment of its evolution. Our study provides molecular data confirming and expanding the hypothesis that the sea urchin larva contains cell types with a pancreatic-like molecular wiring among which neurons are endocrine-like, while the digestive tract contains both a broad spectrum of endocrine-like and well-defined populations of exocrine-like cells. Using single cell transcriptomics and volume EM we demonstrate that both from molecular and morphological perspectives acinar-like cells are homologous to the mammalian exocrine pancreas cells. The validation of the presence of acinar-like cells in a “pancreas-less” non-chordate deuterostome strengthens the hypothesis that the cellular building blocks of pancreas predate the formation of pancreas as a distinct organ in the vertebrate lineage. What were the evolutionary forces that shaped the coalescence of the pancreatic cells into a distinct organ in mammals still remains an open question and needs further investigation. Of great interest is our finding that sea urchin acinar cells express insulin pathway-related genes, such as insulin receptor (*Insr*) and insulin degrading enzyme (*Ide*), suggesting the interaction between endocrine and exocrine cells in sea urchins. Furthermore, both populations are in close connection within the sea urchin stomach region, a feature which is also found in mammalian acinar cells, suggesting a possible evolutionary conserved crosstalk between the insulinergic cells and pancreatic exocrine cells, a phenomenon that would take place later during development when insulin is produced by the posterior gut cells.

As postulated in the past by different authors, tissue level organization enables the coordination of functions between different cells and division of labor. One possibility is that the increase of organismal complexity and physical distances of the different cell types as well as the subsequent increase in homeostatic control and energy costs balance, created the need for inventing a more efficient system in which cells are able to perform their individual tasks in a coordinated and finely controlled manner as is the case of pancreas. The creation of such a system could have been eased by the existence of an already established primitive crosstalk of the individual pancreatic cells, something we believe to be possible, since we found that sea urchin acinar-like cells express genes that could potentially allow them to respond to insulin produced by different endocrine cell types of the digestive tract. In fact, such a crosstalk between endocrine and exocrine pancreas has been demonstrated as early as in 1882, with more recent studies suggesting an intimate regulation of the exocrine pancreas by insulin and potentially other islet cell products (Kühne and Lea, 1882; Czakó et al., 2009; Overton and Mastracci, 2022). Similarly, it has been shown that also the exocrine pancreas is able to control the differentiation of the endocrine β-cells (Overton and Mastracci, 2022). Demonstrating such an interaction was already present in an early branched deuterostome would be of great interest, since it would suggest a crosstalk between the exocrine and endocrine pancreatic cells that would predates (evolutionarily speaking) their spatial co-localization in a distinct organ. Another possibility is that this machinery is, in fact, used to interact with the neighboring sea urchin esophageal cells known to produce a derived insulin-like peptide (Perillo and Arnone 2014).

Taken together we hypothesize that the increase of the distances among the vertebrate digestive tract cell types, a feature directly tied to its increase in cell type content, created the need for diversification of the different functions by specific tissue autonomous units, in this case the pancreas. As a result, pancreatic cell types were brought together to form a distinct organ versus being scattered within the digestive tract of animals to reduce the physical distance of the individual pancreatic cells and to allow a tissue autonomous regulation through paracrine signaling resulting to a precise coordination of cell activities, a faster response and novel functions. A snapshot of this evolutionary process is reflected by the shift of pancreatic cells organization from being scattered within the digestive tract in invertebrates such as echinoderms, cephalochordates and tunicates (Reinecke and Collet, 1998; Olinski et al., 2006; Arda et al., 2013; Lecroisey et al., 2015), to forming simple pancreatic organs with distinct endocrine and exocrine domains in early branching vertebrates such as teleost fish (Falkmer et al., 1985; Yui and Fujita, 1986; Youson et al., 2006), leading to their final interconnection and embedding of the endocrine and exocrine tissues to form a proper pancreas in mammals.

## Data Availability

The datasets presented in this study can be found in online repositories. The names of the repository/repositories and accession number(s) can be found below: https://datadryad.org/stash, https://doi.org/10.5061/dryad.n5tb2rbvz.
