# Supplementary material for: Integrating single cell transcriptomics and volume electron microscopy confirms the presence of pancreatic acinar-like cells in sea urchins
Source: Front Cell Dev Biol. 2022 Aug 19;10:991664. doi: 10.3389/fcell.2022.991664 (PMC9437490; doi:10.3389/fcell.2022.991664)
Supplement: Supplementary file 3 [file Table1.DOCX]

**Supplementary Table 1. Primer sequences used to isolate and amplify the genes of interets.**

| **Gene name** | **Forward primer** | **Reverse primer** |
| --- | --- | --- |
| *Sp-Mnx* | ACCATTGATGCGCTCCTTT | ATTTCTAGCAAATGACTGTTGACG |
| *Sp-Try2* | AATCGTACTCCTCTCCTGCG | ATTGGTTCGGGGCAAAAGTC |
| *Sp-Rfx3* | AGGTTAGGCACAAGGGGAAA | GCAATGTCAGGTCACGGATC |
| *Sp-FoxA* | TCCCACCCCAACCGACTCCG | CGTCCCTTCGAAATGAATGGACAGGG |
