# Supplementary figures and images for: Integrating single cell transcriptomics and volume electron microscopy confirms the presence of pancreatic acinar-like cells in sea urchins
Source: Front Cell Dev Biol. 2022 Aug 19;10:991664. doi: 10.3389/fcell.2022.991664 (PMC9437490; doi:10.3389/fcell.2022.991664)

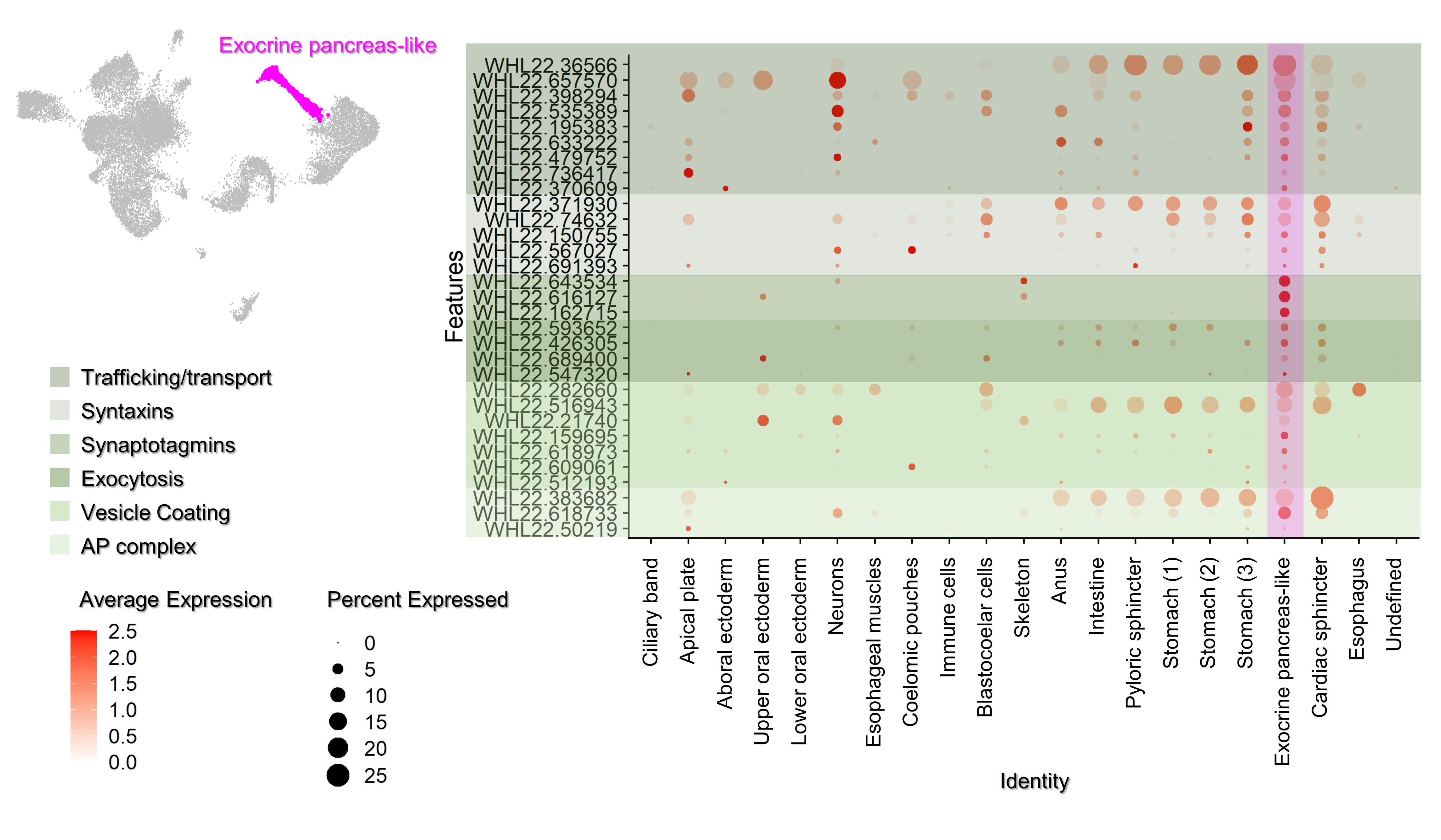

Supplement: Supplementary file 4 [file Image3.TIF]

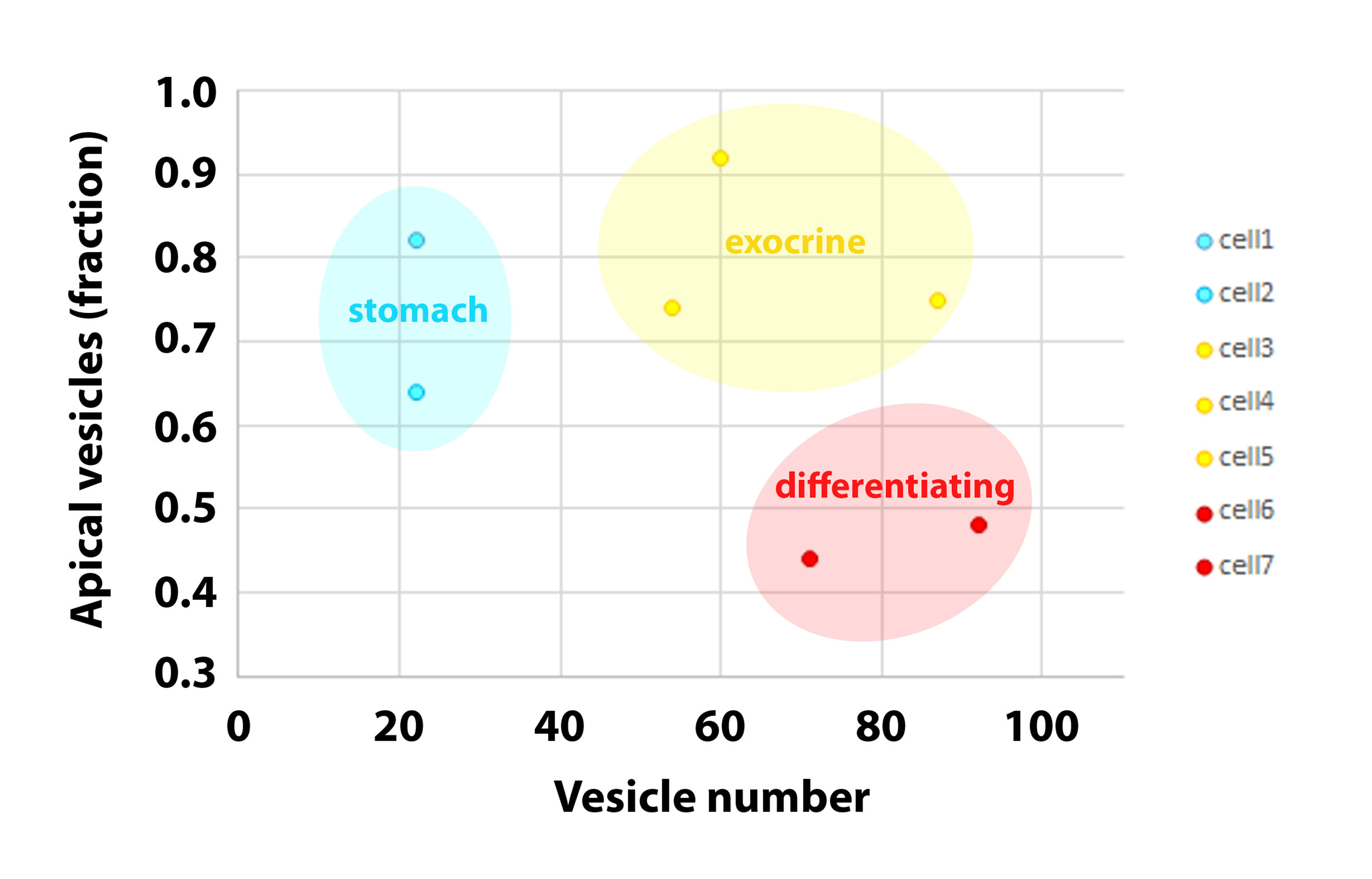

Supplement: Supplementary file 5 [file Image4.TIF]

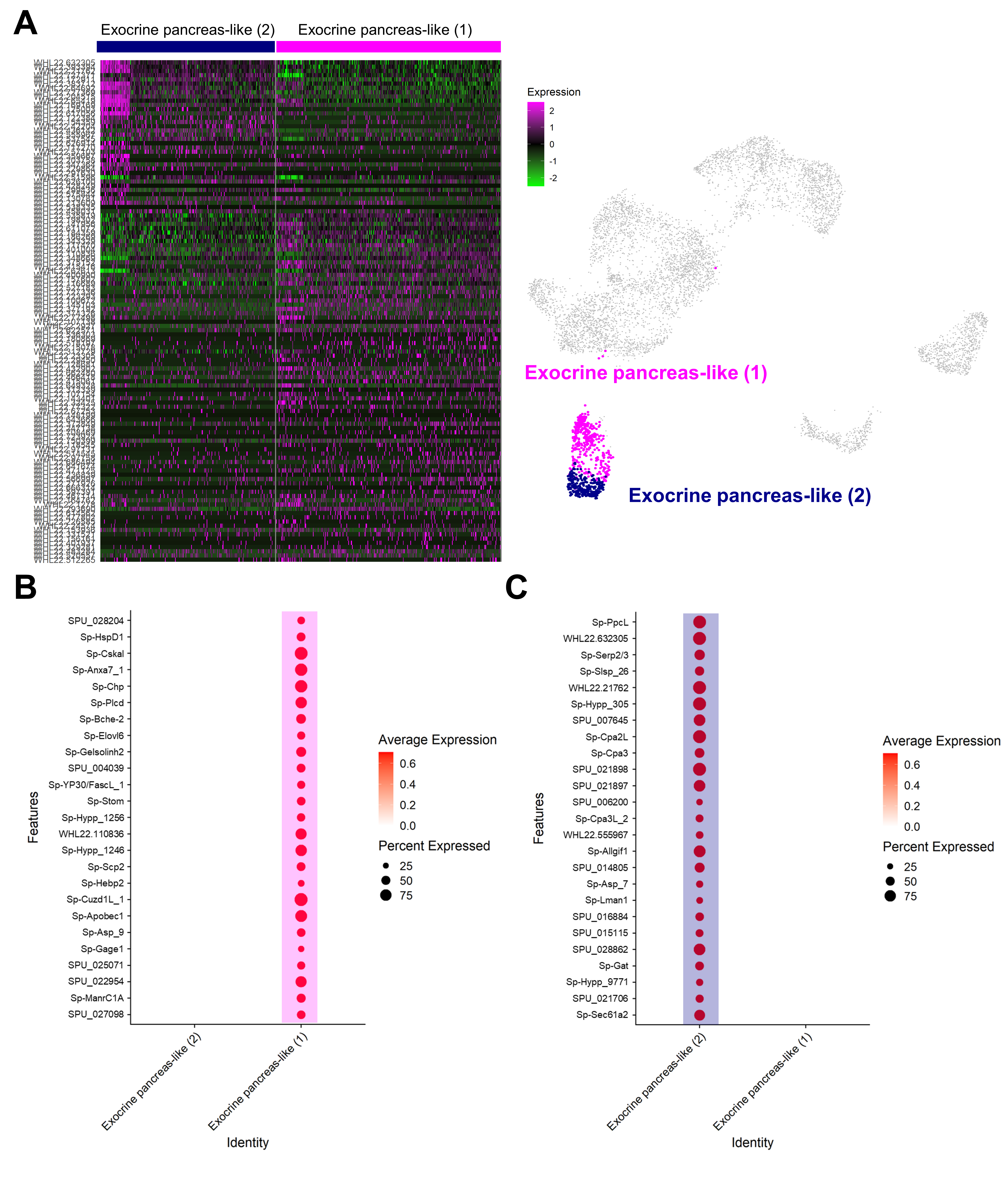

Supplement: Supplementary file 6 [file Image2.TIF]

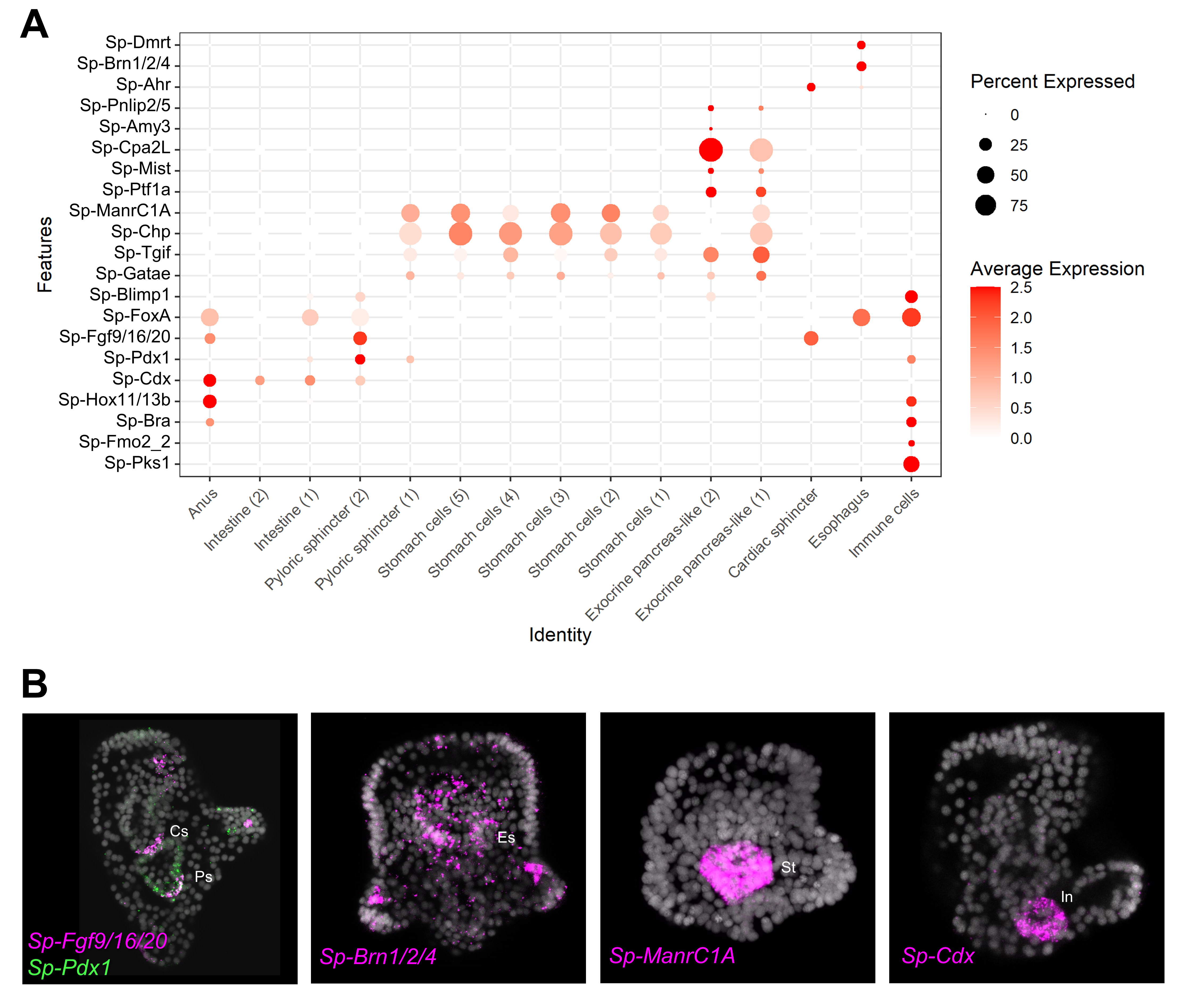

Supplement: Supplementary file 7 [file Image1.TIF]
